# Reduced HDAC2 in skeletal muscle of COPD patients

**DOI:** 10.1186/s12931-017-0588-8

**Published:** 2017-05-19

**Authors:** Masako To, Elisabeth B. Swallow, Kenich Akashi, Kosuke Haruki, S Amanda Natanek, Michael I. Polkey, Kazuhiro Ito, Peter J. Barnes

**Affiliations:** 1 Airway Disease Section, National Heart and Lung Institute, Imperial College, Dovehouse Street, London, SW3 6LY UK; 2 NIHR Respiratory Biomedical Research Unit at the Royal Brompton and Harefield Foundation Trust & Imperial College, London, UK; 3 Department of Laboratory Medicine, Dokkyo Medical University Koshigaya Hospital, 2-1-50 Minami-Koshigaya, Koshigaya-City, Saitama 343-8555 Japan; 4 Molecular Medicine, National Heart and Lung institute, Imperial College London, London, SW7 2AZ UK

**Keywords:** COPD, Skeletal muscle dysfunction, HDAC2, Nuclear factor-kappa B, Apoptosis

## Abstract

**Background:**

Skeletal muscle weakness in chronic obstructive pulmonary disease (COPD) is an important predictor of poor prognosis, but the molecular mechanisms of muscle weakness in COPD have not been fully elucidated. The aim of this study was to investigate the role of histone deacetylases(HDAC) in skeletal muscle weakness in COPD.

**Methods and results:**

Twelve COPD patients, 8 smokers without COPD (SM) and 4 healthy non-smokers (NS) were recruited to the study. HDAC2 protein expression in quadriceps muscle biopsies of COPD patients (HDAC2/β-actin: 0.59 ± 0.34) was significantly lower than that in SM (1.9 ± 1.1, *p* = 0.0007) and NS (1.2 ± 0.7, *p* = 0.029). HDAC2 protein in skeletal muscle was significantly correlated with forced expiratory volume in 1 s % predicted (FEV_1_ % pred) (r_s_ = 0.53, *p* = 0.008) and quadriceps maximum voluntary contraction force (MVC) (r_s_ = 0.42, *p* = 0.029). HDAC5 protein in muscle biopsies of COPD patients (HDAC5/β-actin: 0.44 ± 0.26) was also significantly lower than that in SM (1.29 ± 0.39, *p* = 0.0001) and NS (0.98 ± 0.43, *p* = 0.020). HDAC5 protein in muscle was significantly correlated with FEV_1_ % pred (r_s_ = 0.64, *p* = 0.0007) but not with MVC (r_s_ = 0.30, *p* = 0.180). Nuclear factor-kappa B (NF-κB) DNA binding activity in muscle biopsies of COPD patients (10.1 ± 7.4) was significantly higher than that in SM (3.9 ± 7.3, *p* = 0.020) and NS (1.0 ± 1.2, *p* = 0.004and significantly correlated with HDAC2 decrease (r_s_ = −0.59, *p* = 0.003) and HDAC5 (r_s_ = 0.050, *p* = 0.012). HDAC2 knockdown by RNA interference in primary skeletal muscle cells caused an increase in NF-κB activity, NF-κB acetylation and basal tumour necrosis factor (TNF)-α production, as well as progressive cell death through apoptosis.

**Conclusion:**

Skeletal muscle weakness in COPD may result from HDAC2 down-regulation in skeletal muscle via acetylation and activation of NF-κB. The restoration of HDAC2 levels might be a therapeutic target for improving skeletal muscle weakness in COPD.

**Electronic supplementary material:**

The online version of this article (doi:10.1186/s12931-017-0588-8) contains supplementary material, which is available to authorized users.

## Background

Chronic obstructive pulmonary disease (COPD) is an increasing health problem and it is predicted to be the third most common cause of death worldwide by 2020 [1]. In COPD patients, skeletal muscle weakness is an extra-pulmonary manifestation that markedly reduces their quality of life and survival. Quadriceps weakness [2–4] and a decrease in quadriceps endurance [5, 6] have been reported in patients with COPD. Skeletal muscle strength in COPD, measured by maximum voluntary contraction (MVC), is lower in approximately a third of all COPD patients than in age-matched controls [7]. Quadriceps strength is a better predictor of mortality in COPD than FEV_1_ in patients with moderate to very severe lung function impairment [8].

Skeletal muscle in COPD shows cellular structural changes, including a reduction in type I fibres, fibre cross-sectional area and capillary contacts to muscle fibres [9], several metabolic changes and activation of the proinflammatory transcription factor nuclear factor-kappa B (NF-κB) [10], mitochondrial dysfunction [11] and enhanced autophagy [12]. Recently, it was reported that the histone deacetylases (HDAC) 3 and HDAC4 might be associated with muscle dysfunction in COPD [13, 14]. These reports were descriptive and failed to demonstrate the precise molecular relationship between HDAC reduction and muscle dysfunction. Furthermore, data suggesting the potential molecular mechanisms of depression of HDACs in COPD muscle were not shown. The precise molecular mechanisms of muscle weakness underlying COPD need to be elucidated.

HDAC are a family of enzymes that remove acetyl groups from amino acids, usually lysine residues, and modify inflammatory gene expression by regulating histone acetylation and chromatin structure as well as through non-histone protein acetylation. There are 11 isoforms of Type I and II HDACs [15]. We have previously shown that total HDAC activity is reduced in peripheral lung and alveolar macrophages from COPD patients, and that the reduction correlates with the degree of airflow limitation [16]. In particular, the protein levels and mRNA levels of HDAC2 and HDAC5 mRNA levels were reduced in peripheral lung obtained from COPD patients [16].

HDACs also target non-histone proteins, including transcription factors such as NF-κB, and a reduction of HDAC induces hyperacetylation of NF-κB. NF-κB is activated in lung epithelial cells and macrophages of COPD patients and regulates the increased expression of proinflammatory cytokines, such as tumour necrosis factor(TNF)-α, interleukin(IL)-1β and IL-6, all of which are increased in COPD patients. NF-κB activity is critically regulated by post-translational modifications, including acetylation. Acetylation of the p65 component of NF-κB at lysine^221^ abolishes binding of p65 to inhibitor of NF-κB-β (IκBβ) [17], whereas acetylation of p65 at lysine^310^ is necessary for transcriptional activation [17].

Based on our previous report that HDAC2 and HDAC5 levels were decreased in peripheral lung from COPD patients [16], we hypothesised that HDAC2 and/or 5 protein expression in skeletal muscle from COPD patients was also reduced and this reduction is associated with skeletal muscle weakness in COPD patients. The aim of this study was to explore the molecular mechanisms of muscle weakness in COPD patients using clinical samples and a skeletal muscle cell line. Understanding the molecular mechanisms of skeletal muscle weakness in COPD may lead to new therapeutic approaches to this disabling problem.

## Methods

### Patient recruitment

Patients and healthy subjects were recruited from the Outpatient Department of the Royal Brompton Hospital. COPD patients were diagnosed and classified using Global Initiative for Obstructive Lung Disease (GOLD) criteria [18]. Patients and controls were free of relevant co-morbidities, such as neurological or cardiac disease, which might cause skeletal muscle weakness as were those with co-morbidities known to cause systemic inflammation; however circulating markers of inflammation were not measured. An exacerbation requiring a change in medical therapy in the preceding 12 weeks was also an exclusion criteria. Biopsies were obtained from two studies (Brompton, Harefield &NHLI Ethics Committee, Ref 03-148) and (North London REC 3, Ref 06/Q0410/54) and written informed consent was obtained from all subjects.

Skeletal muscle strength was evaluated using maximal voluntary contraction force (MVC). MVC is one of the methods to evaluate muscle weakness which is recognised and described in the recent ERS/ATS statement [11]. It is used routinely in our laboratory and as previously published articles [7].

### Skeletal muscle biopsy

Percutaneous biopsy of the vastus lateralis was performed using the technique of Bergstrom [19] after subjects had rested for 20 min, on a day without strenuous physical activity. Samples for mRNA and protein analysis were frozen in liquid nitrogen, prior to storing at -80 °C.

### Protein extraction from skeletal muscle biopsies

Frozen skeletal muscle biopsy specimens were crushed and ground in liquid nitrogen with a pestle and mortar. Whole cell extraction was performed using the Nuclear Extraction kit (Active Motif, Carlsbad, CA) according to the manufacturers’ instructions with minor modification. The protein concentration of each sample was determined with Bradford Bio-Rad Protein Assay (Bio-Rad Laboratories, Hertford, UK) using bovine serum albumin as a standard.

### NF-κB DNA binding assay

NF-κB DNA binding activity was measured using TransAM NFκB p65 Activation Assay kit (Active Motif) according to the manufacturers’ protocol. A standard curve for relative activity was generated using a recombinant NF-κB p65 protein. The relative activity of the top standard was defined to be 100 and the relative activity of each sample determined using the standard curve. Results were standardized according to the protein content (μg) in each sample determined as described above.

### Quantitative real-time polymerase chain reaction (qRT-PCR)

Total ribonucleic acid (RNA) extraction, reverse transcription of total RNA and QRT-PCR were performed as previously shown [16]. The amount of target transcript in each sample was standardized against levels of GNB2L1.

### Gel electrophoresis and western blotting

Whole cell extracts (30 μL each) were electrophoresed and protein samples in the gels were transferred to a nitrocellulose membrane as previously shown [16]. Immunoreactive bands of HDAC2, HDAC5 and β-actin were detected with anti-HDAC2 antibody (Santa Cruz Biotechnology, Santa Cruz, CA), anti-HDAC5 antibody (Santa Cruz Biotechnology) and anti-β actin antibody (Abcam, Cambridge, UK), respectively. Band densities of HDAC2 and HDAC5 in each sample were normalized with that of the β-actin or glyceraldehyde 3-phosphate dehydrogenase (GAPDH).

### RNA interference

Primary normal human skeletal muscle cells were purchased from Lonza Walkersville Inc. (Walkersville, MD) and grown in Skeletal Muscle Cell Medium BulletKit^TM^. Cells were cultured in 6-well plates at a density of 1 × 10^6^ cells/well, and transfected with 100nM of HDAC2 siRNA (Life Technologies) using PrimeFect^TM^ siRNA transfection reagent (Lonza Walkersville Inc.) according to the manufacturers’ instructions.

### Caspase 3 activity assay

The activity of caspase-3 was determined using a commercial Caspase-3 Colorimetric Assay Kit (BioVision Inc., Milpitas, CA). Fifty μL of chilled Cell Lysis Buffer was applied to each well of a 24-well plate, and cells were incubated on ice for 10 min. After collection of cell lysis supernatant, the cell lysate (100 μg of protein) was added to 50 μl Reaction Buffer followed by addition of the substrate for each sample and incubated at 37 °C for 1–2 h. Colorimetric determination was performed by a microtiter plate reader.

### Statistical analysis

Results were represented as mean ± standard deviation of the mean. Multiple comparisons were performed by Kruskal-Wallis analysis, followed by post-test. Comparisons between two groups were performed with non-parametric Mann Whitney *U*-test. Correlation analysis was performed with Spearman's rank correlation coefficient. A *p* value < 0.05 was considered statistically significant. Analyses were performed using Graph Pad Prism 4 Software (Graph Pad Prism, San Diego, CA).

## Results

### Subjects

A total of 24 subjects (12 COPD patients aged 66 ± 6 years, 8 smokers without COPD (SM) aged 67 ± 9 years, and 4 healthy non-smokers (NS) aged 63 ± 10 years) were recruited for this study (Table 1). FEV_1_ % predicted of COPD patients (33 ± 11%) showed significant airflow limitation compared to both NS (104 ± 15%) and SM (107 ± 20%). One COPD patients were GOLD2, 4 patients GOLD3 and 7 patients GOLD4 category. Eleven out of 12 COPD patients were ex-smokers and only one patient was a current smoker, whereas 2 of 8 smokers without COPD were current smokers. Ten out of 12 COPD patients were treated with inhaled long-acting beta_2_-agonists. Two out of 12 COPD patients were treated with inhaled corticosteroids but none with systemic corticosteroids within 3 months of the study. One out of 12 COPD patients was treated with low dose oral theophylline.

**Table 1:** Patient Characteristics

	Non smokers	Smokers without COPD	COPD
N	4	8	12
Age (yr)	63 ± 10	67 ± 9	66 ± 6
Sex (F:M)	1:3	2:6	0:15
Smoking status (current/ex/never)	0/0/4	2/6/0	1/11/0
Pack-year	0 ± 0	16 ± 22	40 ± 23
BMI (kg/m^2^)	23 ± 2	30 ± 7	25 ± 5
FEV_1_ (L)	3.00 ± 0.91	2.91 ± 0.41	1.05 ± 0.37^a,^ ^b^
FEV (%predicted)	104 ± 15	107 ± 20	33 ± 11 ^a,^ ^b^
RV %predicted (%)	ND	ND	207 ± 40
KCO %predicted (%)	ND	ND	52 ± 19
MVC (kg)	40 ± 19	41 ± 13	34 ± 10
PaO_2_ (kPa)	ND	ND	8.7 ± 2.2
Systemic Steroids	NA	NA	0
ICS	NA	NA	5

*Abbreviations*: *BMI* body mass index, *FEV*_*1*_ forced expiratory volume in 1 s, *RV* residual volume, *KCO* carbon monoxide transfer coefficient, *MVC* maximum voluntary contraction, *PaO*_*2*_ partial pressure of oxygen in arterial blood, *ICS* inhaled corticosteroid, *COPD* chronic obstructive pulmonary disease, *ND* not determined, *NA* not applicable

^a^ *p* < 0.01 compared to NS; ^b^ *p* < 0.01 compared to SM

### Expression of HDAC protein in skeletal muscle

**Fig. 1:**
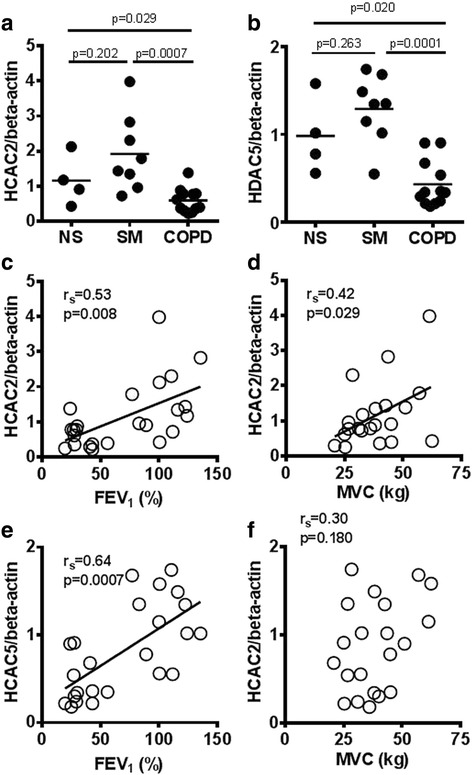
HDACs in skeletal muscle biopsies and their correlations with clinical parameters. Whole cell proteins were extracted from quadriceps muscle biopsy specimens. HDAC2 protein (**a**) and HDAC5 protein (**b**) were measured by sodium dodecyl sulphate polyacrylamide gel electrophoresis/Western blot. Correlations between HDACs in skeletal muscle and clinical parameters were evaluated. FEV_1_ %predicted *vs* HDAC2 protein expression (**c**), MVC *vs* HDAC2 protein expression (**d**), FEV_1_ %predicted *vs* HDAC5 protein expression (**e**), MVC *vs* HDAC5 protein expression (**f**). *Abbreviations*: healthy non-smokers (*NS*), smokers without COPD (*SM*), chronic obstructive pulmonary disease (*COPD*), histone deacetylase (*HDAC*), forced expiratory volume in 1 s (*FEV*_*1*_); maximum voluntary contraction (*MVC*)

The level of HDAC2 protein in skeletal muscle of COPD patients (HDAC2/β-actin ratio: 0.59 ± 0.34) was significantly lower than that SM (1.9 ± 1.1, *p* = 0.0007) and NS (1.2 ± 0.7, *p* = 0.029; Fig. 1a). HDAC5 protein in skeletal muscle of COPD patients (0.44 ± 0.26) was also significantly lower than that of SM (1.29 ± 0.39, *p* = 0.0001) and NS (0.98 ± 0.43, *p* = 0.020; Fig. 1b). In contrast, there were no significant differences in gene transcription levels (mRNA) of HDAC2 and HDAC5 in skeletal muscles among the 3 subject groups (Additional file 1: Figure S1).

### Associations between skeletal muscle HDAC expression and clinical parameters

HDAC2 protein levels in skeletal muscle significantly correlated with both FEV_1_ % predicted (r_s_ = 0.53, *p* = 0.008; Fig. 1c) and quadriceps MVC (r_s_ = 0.42, *p* = 0.029; Fig. 1d). HDAC5 protein in skeletal muscle correlated with FEV_1_ % predicted (r_s_ = 0.64, *p* = 0.0007; Fig. 1e), whereas it did not correlate with MVC (r_s_ = 0.30, *p* = 0.180; Fig. 1f). HDAC2 was also significantly correlated with K_CO_ % predicted (r_s_ = 0.67, *p* = 0.027) and TL_CO_% predicted (r_s_ = 0.50, *p* = 0.044), whereas HDAC5 did not (Table 2).

**Table 2:** Correlation between clinical parameters and HDAC2, HDAC5 and NF-κB DNA binding activity in all subjects

	HDAC2	HDAC5	NF-κB
Age	0.04	−0.05	0.01
Pack-year	−0.28	−0.35	0.62**
BMI (kg/m^2^)	0.36	0.04	−0.03
FEV_1_%predicted	0.53**	0.64**	−0.54**
FEV_1_/FVC %	0.55**	0.68**	−0.53**
RV % predicted	−0.33	−0.33	0.59*
KCO % predicted	0.67*	0.03	−0.74*
TL_CO_% predicted	0.50*	0.43	−0.81**
PaO_2_	0.32	0.39	−0.54*
PaCO_2_	−0.16	−0.07	0.30
MVC	0.42*	0.30	−0.50*
NF-κB	−0.59**	−0.50*	NA
HDAC 2 protein	NA	0.63**	−0.59**
HDAC5 protein	0.63**	NA	−0.50*
HDAC1 mRNA	−0.09	−0.02	0.13
HDAC2 mRNA	0.14	−0.11	−0.27
HDAC5 mRNA	−0.36	−0.44	0.12

*Abbreviations*: *BMI* body mass index, *FEV*_*1*_ forced expiratory volume in 1 s, *RV* residual volume, *KCO* carbon monoxide transfer coefficient, *TL*_*CO*_ transfer factor for carbon monoxide in the lung, *MVC* maximum voluntary contraction, *PaO*_*2*_ partial pressure of oxygen in arterial blood, *NF-κB* nuclear factor-kappa B, *HDAC* histone deacetylase, *NA* not applicable

*p < 0.05; ** *p* < 0.01

### NF-κB DNA binding activity in skeletal muscle

**Fig. 2:**
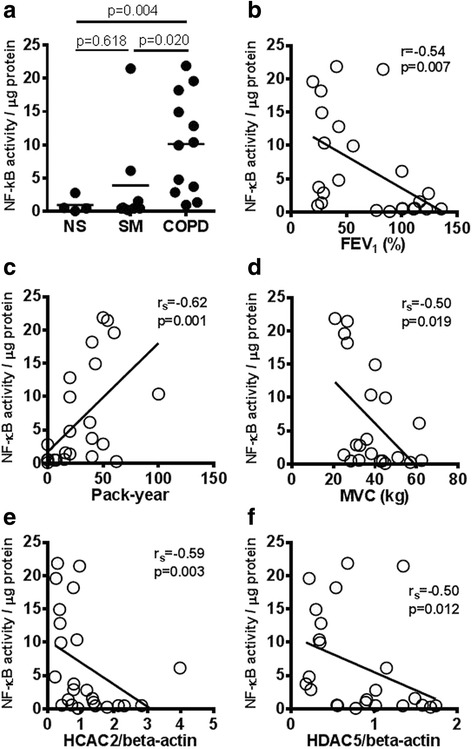
NF-κB DNA binding activity in skeletal muscle biopsy specimens. **a** NF-κB DNA binding activity was measured in whole cell extracts from skeletal muscle biopsies. Correlation between NF-κB DNA binding activity and FEV_1_ %predicted (**b**), cigarette exposure in pack-years (**c**). HDAC2 protein expression (**d**) and HDAC5 protein expression (**e**). Abbreviations: healthy non-smokers (*NS*), smokers without COPD (*SM*), chronic obstructive pulmonary disease (*COPD*), nuclear factor-kappa B (NF-κB), forced expiratory volume in 1 s (*FEV*_*1*_), maximum voluntary contraction (*MVC*)

NF-κB DNA binding activity in skeletal muscle of COPD (10.1 ± 7.4, (relative activity/μg protein)) was significantly higher than that of NS (1.0 ± 1.2, *p* = 0.004) and SM (3.9 ± 7.3, *p* = 0.020) (Fig. 2a). NF-κB DNA binding activity correlated negatively with FEV_1_ % predicted (r_s_ = -0.54, *p* = 0.007; Fig. 2b), positively with pack-years (r_s_ = 0.62, *p* = 0.001; Fig. 2c) and negatively with MVC (r_s_ = 0.50, *p* = 0.019; Fig. 2d). NF-κB DNA binding activity also correlated with KCO % predicted (r_s_ = -0.74, *p* = 0.011), RV % predicted (r_s_ = 0.58, *p* = 0.023) and TL_CO_% predicted (r_s_ = −0.81, *p* = 0.0002) (Table 2). In addition, NF- κB DNA binding activity in skeletal muscle significantly correlated with HDAC2 protein expression (r_s_ = −0.59, *p* = 0.003, Fig. 2e) and HDAC5 protein expression (r_s_ = −0.50, *p* = 0.012, Fig. 2f).

### HDAC2 knockdown induced NF-κB activation in skeletal muscle cells

**Fig. 3:**
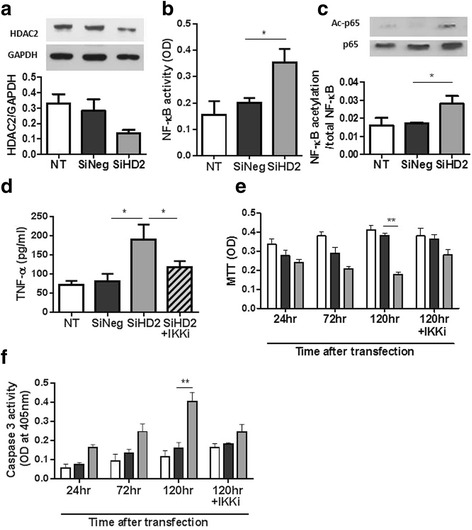
Impact of HDAC2 knockdown on NF-κB activation in primary skeletal muscle cells. Effect of HDAC2 RNA interference on HDAC2 protein (**a**), NF-κB activity (**b**), NF-κB -p65 acetylation at lysine^310^ (**c**), basal TNF-α production (**d**) at 24 h after transfection, cell number determined by MTT assay (**e**) and cell apoptosis evaluated with caspase 3 activity assay (**f**) in primary skeletal muscle cells. IKK inhibitor (IKKi), SC514, was given 12 h after transfection. *: *p* < 0.05, **: *p* < 0.01. *Abbreviations*: nuclear factor-kappa B (*NF-κB*), histone deacetylase (*HDAC*), glyceraldehyde 3-phosphate dehydrogenase (*GAPDH*), no transfection (*NT*), negative control short interfering RNA (*SiNeg*), short interfering RNA of HDAC2 (*SiHD2*); IκB kinase (IKK), optical density (*OD*), acetylated p65 (*Ac-p65*), methylthiazol tetrazolium (MTT:)

In primary skeletal muscle cells, transfection of HDAC2 siRNA induced a 73% reduction in HDAC2 mRNA evaluated by RT-PCR (data not shown) and a 52% reduction in HDAC2 protein evaluated by Western blotting (Fig. 3a) at 24 h after transfection. The basal NF-κB activity in nuclear extracts (40 μg) was significantly higher in HDAC2 knockdown cells than in negative control siRNA (SiNeg) transfected cells and non-transfected cells (NT): (SiHDAC2 0.35 ± 0.053 OD; NT 0.15 ± 0.054; SiNeg: 0.20 ± 0.028, *p* < 0.05, *n* = 3; Fig. 3b). The acetylation of Lys^310^ p65-NF-κB was also elevated in HDAC2 knockdown cells (ratio of acetylated p65/total p65: NT: 0.016 ± 0.0041, SiNeg: 0.17 ± 0.00056, SiHDAC2: 0.28 ± 0.0043 OD, *p* < 0.05, *n* = 3; Fig. 3c). The basal level of TNF-α increased in the supernatants of HDAC2 knockdown cells, and TNF-α production was inhibited by SC514 (20 μM), an IKK-β inhibitor, when given 12 h after transfection (Fig. 3d). The skeletal muscle cell viability determined by methylthiazol tetrazolium (MTT) assay decreased over time after HDAC2 siRNA transfection, whereas SiNeg transfection did not affect viability (Fig. 3e). More importantly, the reduction in skeletal muscle cell viability seen 120 h after transfection was inhibited by treatment with the IKK-β inhibitor. Skeletal muscle cell apoptosis evaluated by caspase 3 activity in HDAC2 knockdown cell (120 h after transfection) was also significantly higher than controls (*p* < 0.01, Fig. 3f). The elevated apoptosis in HDAC2 knockdown cells were also attenuated by IKK inhibitor.

## Discussion

In this study, we have demonstrated for the first time a reduction in HDAC2 protein in skeletal muscle from COPD patients, and this is of a similar order to the reduction in HDAC2 protein expression previously reported in peripheral lung tissue, alveolar macrophages, sputum macrophages, bronchial biopsies and peripheral blood mononuclear cells from COPD patients [16, 20]. HDAC2 protein expression in skeletal muscle was correlated with lung function (Fig. 1c), as previously shown in peripheral lung [16], but more importantly HDAC2 protein levels also correlated well with quadriceps weakness as measured by MVC (Fig. 1d). This suggests that decreased HDAC2 may be involved in the skeletal muscle weakness seen in COPD patients. In fact, HDAC2 knockdown by RNA interference decreased the viability of skeletal muscle cells in vitro through induction of apoptosis, as shown in Fig. 3e and f.

Oxidative stress resulting from cigarette smoke extracts, is likely to be a major cause of the reduction in HDAC2 [21, 22]. There is compelling evidence for increased oxidative stress in skeletal muscle of COPD patients and a relationship with muscle weakness. For example, lipid peroxidation and nitrotyrosine are increased in skeletal muscle of COPD patients [23] and muscle protein carbonylation as a result of oxidative stress is associated with quadriceps weakness in COPD [24]. Barreiro et al. also demonstrated a high level of oxidative stress in skeletal muscle in COPD (carbonylation of proteins), which is associated with muscle dysfunction [25]. Furthermore, treatment with an anti-oxidant (*N*-acetyl cysteine) increases exercise endurance time compared to a placebo [26]. In addition, Rossman and colleagues demonstrated an association between systemic oxidative stress and skeletal muscle dysfunction in COPD patients and also showed that systemic infusion of ascorbate, an antioxidant, induced resistance to muscle fatigue in COPD patients [27]. Thus, systemic oxidative stress is associated with skeletal muscle dysfunction and the reduction of HDAC2 in skeletal muscle in COPD patients. Moreover, HDAC2 depletion is known to suppress nuclear factor erythroid 2-related factor (Nrf2), a redox-sensitive transcription factor that induces the expression of multiple anti-oxidant genes, resulting in impaired anti-oxidant defenses [28], and an increase in endogenous oxidative stress. Thus, HDAC2 repression and oxidative stress induction may enter into a vicious perpetuating circle.

NF-κB DNA binding activity is significantly activated in skeletal muscle from COPD patients, as previously reported in a small study of cachectic COPD patients [10]. As NF-κB is an oxidative stress-sensitive transcription factor, increased oxidative stress in skeletal muscle might the cause of NF-κB activation [29, 30]. In this study, we have also demonstrated a correlation between HDAC2 expression and NF-κB DNA binding activity in skeletal muscle cells (Fig. 2d). HDAC2 knockdown in primary skeletal muscle cells also increased NF-κB DNA binding (Fig. 3b). NF-κB is recognized to be an acetylated protein [17] and its acetylation status controls its activity. HDAC3, sirtuin-1 and sirtuin-2 have previously been shown to deacetylate p65-NF-κB at Lys^310^ [31–33]. Although HDAC2 over-expression is reported to be insufficient to induce Lys^310^deacetylation [32], based on our current data, we believe that at least in part, HDAC2 suppression causes hyperacetylation of NF-κB, leading to increased NF-κB activity and increased release of inflammatory cytokines, such as TNF-α.

**Fig. 4:**
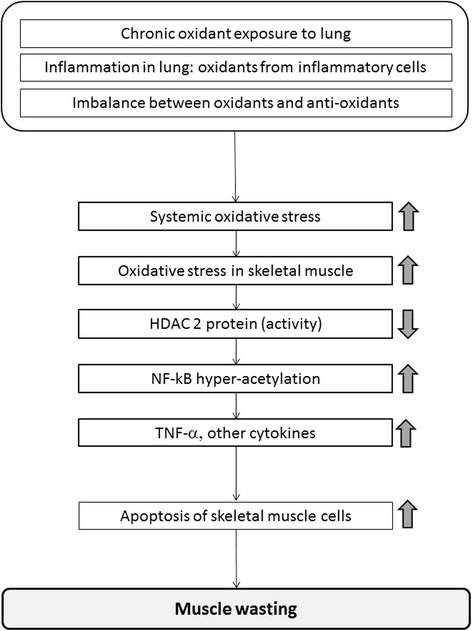
A hypothetical scheme of how oxidative stress induces skeletal muscle dysfunction in COPD. HDAC2 reduction caused by oxidative stress in COPD (systemic and local) increase NF-kB activation and TNF-α expression resulting in muscle cell apoptosis and subsequent skeletal muscle dysfunction

Thus reduced HDAC2 and activation of NF-κB are potentially involved in skeletal muscle weakness, but the molecular mechanisms are unclear. The role of HDAC2 in proliferation and differentiation of skeletal muscle are controversial. Muscle RING-Finger Protein-1 (MuRF1) is an E3 ubiquitin-protein ligase that induces muscle atrophy in a rodent model, and is regulated by the NF-κB pathway [34]. Mice constitutively expressing active IKK-β and mice with IκB inhibition showed elevated MuRF1 expression and muscle atrophy [34]. However, reports of MuRF1 expression in the quadriceps of COPD patients have not consistently shown elevated expression [35–37]. Furthermore, HDAC inhibition by trichostatin A is reported to inhibit unloaded-induced muscle atrophy through elevation of MuRF1 expression [38]. However, this may not be analogous to the skeletal muscle weakness in COPD patients. On the other hand, we have demonstrated that HDAC2 knockdown increased basal TNF-α production concomitant with NF-κB activation in primary skeletal muscle cells. TNF-α production in HDAC2 knockdown cells was also inhibited by an IKK-β inhibitor (Fig. 3d). TNF-α plays an important role in the development of muscular abnormalities, resulting in loss of muscle mass and function [39, 40]. Both skeletal muscle cell viability determined by MTT assay and apoptosis evaluated by caspase 3 activity were markedly decreased in HDAC2 knockdown cells (Fig. 3e and f) and reversed by the IKK inhibitor. It is likely that skeletal muscle cells died due to NF-κB activation, potentially through TNF-α production (Fig. 4). HDAC2 reduction might induce apoptosis as HDAC2 knockdown induced apoptosis of skeletal muscle cells in our experiment. In fact, HDAC2 is reported to regulate skeletal muscle homeostasis in mice [41].

HDAC2 is known to be regulated differently in different stages of disease or in different tissue as follows; firstly, reduced enzyme activity can occur without any change of protein or mRNA expression, most likely due to post-translational modifications, such as nitration, oxidation and phosphorylation by oxidative stress. Secondly, HDAC2 protein reduction can occur without any change of transcription-mRNA due to degradation by the proteasome [42, 43]. Thirdly, HDAC2 protein reduction can occur with reduced transcription, particularly seen in more severe COPD or in vitro after very high levels of oxidative stress exposure. In our data, the level of gene transcription HDAC2 (mRNA) was not decreased in COPD skeletal muscle while HDAC2 protein expression was decreased compared with controls. Therefore, it seems to be the second mechanism, with increased HDAC2 protein degradation, as a result of proteasome activation that predominates in skeletal muscle of COPD patients. Unfortunately, the samples from biopsy were too small to evaluate post-translational modification or the activity/molecule after immunoprecipitation. Any reduction in HDAC2 protein will necessarily also reduce its enzyme activity.

Collectively, our data and previously published findings suggest that oxidative stress, which derived originally from lung, then systemic and latterly in skeletal muscle, causes HDAC2 reduction. Then, the reduced HDAC2 leads an increase in NF-kB activation and TNF-α expression resulting in muscle cell apoptosis and subsequent skeletal muscle dysfunction (Fig. 4).

However, there are some limitations in this study. Firstly, some measurements were not possible due to the small size of the biopsies, which were insufficient to analyse all desired parameters, such as HDAC2 activity, oxidative stress level and apoptosis and also a lack of serum samples to evaluate systemic oxidative stress. However, at least, apoptosis of skeletal muscle has previously been reported to be increased in COPD patients compared with controls [44, 45]. Secondly, due to the small sample size, differences of HDAC2/5 in GOLD severity of the COPD patients, drug treatment and smoking status (current smokers or Ex-smokers), which might influence the results, was could not be evaluated. Thirdly, some clinical data including BODE score and fat free mass was not available. A study with larger sample size will be required to confirm our results. It would also be interesting to evaluate the effect of an HDAC activator, such as low dose oral theophylline, on skeletal muscle function in COPD patients with muscle weakness. HDAC5, as well as HDAC2, is reduced in peripheral lung of COPD patients [16]. In the current study, HDAC5 expression in COPD skeletal muscle was also found to be reduced compared with normal smokers, and was correlated with FEV_1_ and FEV_1_/FVC, but not with MVC (Table 2). At least, from the current findings, HDAC2 reduction appears to be more important for the aetiology of muscle weakness in COPD than HDAC5 reduction. Further studies will be needed to explore the association between reduced HDAC5 and skeletal muscle dysfunction in COPD.

## Conclusions

Reduction in HDAC2 in skeletal muscle appears to be involved in skeletal muscle dysfunction in COPD via acetylation and activation of NF-κB. Restoration of HDAC2 expression might be an effective therapeutic target to improve skeletal muscle weakness in COPD patients.

## Additional file

Additional file 1: Figure S1. HDACs mRNA expressions in skeletal muscle biopsies. (JPG 61 kb)

## Abbreviation

COPD: Chronic obstructive pulmonary disease
GAPDH: Glyceraldehyde 3-phosphate dehydrogenase
GNB2L1: Guanine nucleotide-binding protein, beta-2-like 1
GOLD: The Global Initiative for Obstructive Lung Disease
HAT: Histone acetyltransferase
HDAC: Histone deacetylase
IKK: IκB kinase
MuRF1: Muscle RING-Finger Protein-1
MVC: Maximum voluntary contraction
NF-κB: Nuclear factor-kappa B
NrF2: Nuclear factor erythroid 2-related factor
PCR: Polymerase chain reaction
RNA: Ribonucleic acid
SDS/PAGE: Sodium dodecyl sulphate poly-acrylamide gel electrophoresis
siRNA: Short interfering RNA
