# Supplementary figures and images for: Reduced HDAC2 in skeletal muscle of COPD patients
Source: Respir Res. 2017 May 19;18:99. doi: 10.1186/s12931-017-0588-8 (PMC5438490; doi:10.1186/s12931-017-0588-8)

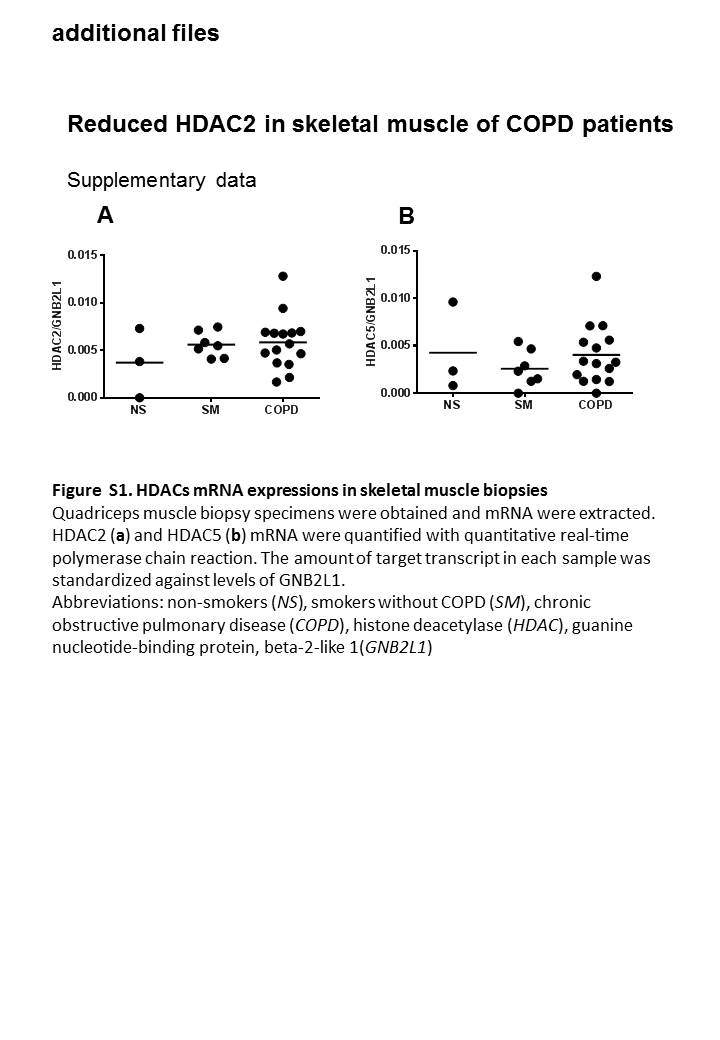

Supplement: Additional file 1: Figure S1. — HDACs mRNA expressions in skeletal muscle biopsies. (JPG 61 kb) [file 12931_2017_588_MOESM1_ESM.jpg]
